# Comparison of Navigation-Related Brain Regions in Migratory versus Non-Migratory Noctuid Moths

**DOI:** 10.3389/fnbeh.2017.00158

**Published:** 2017-09-04

**Authors:** Liv de Vries, Keram Pfeiffer, Björn Trebels, Andrea K. Adden, Ken Green, Eric Warrant, Stanley Heinze

**Affiliations:** ^1^Lund Vision Group, Department of Biology, Lund University Lund, Sweden; ^2^Department of Biology, Marburg University Marburg, Germany; ^3^New South Wales National Parks and Wildlife Service Jindabyne, NSW, Australia

**Keywords:** central complex, Bogong moth, standard brain, 3D-neuroanatomy, migration, navigation, insect brain

## Abstract

Brain structure and function are tightly correlated across all animals. While these relations are ultimately manifestations of differently wired neurons, many changes in neural circuit architecture lead to larger-scale alterations visible already at the level of brain regions. Locating such differences has served as a beacon for identifying brain areas that are strongly associated with the ecological needs of a species—thus guiding the way towards more detailed investigations of how brains underlie species-specific behaviors. Particularly in relation to sensory requirements, volume-differences in neural tissue between closely related species reflect evolutionary investments that correspond to sensory abilities. Likewise, memory-demands imposed by lifestyle have revealed similar adaptations in regions associated with learning. Whether this is also the case for species that differ in their navigational strategy is currently unknown. While the brain regions associated with navigational control in insects have been identified (central complex (CX), lateral complex (LX) and anterior optic tubercles (AOTU)), it remains unknown in what way evolutionary investments have been made to accommodate particularly demanding navigational strategies. We have thus generated average-shape atlases of navigation-related brain regions of a migratory and a non-migratory noctuid moth and used volumetric analysis to identify differences. We further compared the results to identical data from Monarch butterflies. Whereas we found differences in the size of the nodular unit of the AOTU, the LX and the protocerebral bridge (PB) between the two moths, these did not unambiguously reflect migratory behavior across all three species. We conclude that navigational strategy, at least in the case of long-distance migration in lepidopteran insects, is not easily deductible from overall neuropil anatomy. This suggests that the adaptations needed to ensure successful migratory behavior are found in the detailed wiring characteristics of the neural circuits underlying navigation—differences that are only accessible through detailed physiological and ultrastructural investigations. The presented results aid this task in two ways. First, the identified differences in neuropil volumes serve as promising initial targets for electrophysiology. Second, the new standard atlases provide an anatomical reference frame for embedding all functional data obtained from the brains of the Bogong and the Turnip moth.

## Introduction

Every brain is optimized to generate the behavior required for an animal’s survival. As neural tissue is energetically extremely costly, brains have evolved to extract required sensory information in the most economical way, while at the same time guiding behaviors as efficiently as possible (Laughlin, 2001; Niven and Laughlin, 2008). Through evolution, neural circuits have thus been adapted to match each species’ ecological niche, i.e., the combination of behavioral strategy and sensory environment. Uncovering the ways these adaptations have been achieved in different animals will provide major insights into the functional outline of brains in general. While circuit adaptations to sensory requirements have been documented in several species (e.g., el Jundi et al., 2015; Stöckl et al., 2016b), the questions of how behavioral strategies are manifested in the neuroarchitecture of brains remains largely unanswered.

As neural circuits consist of thousands of neurons and span multiple brain regions, it is difficult to examine them directly and in full detail across many animals, even when focusing on the comparably simple brains of insects. In order to pinpoint promising regions of the brain in which species show differential investment in neural tissue, volumes of brain regions have served as a beacon (e.g., Gronenberg and Hölldobler, 1999; Kondoh et al., 2003; Ott and Rogers, 2010; O’Donnell et al., 2013). Volumetric analysis between closely related species inhabiting different environments has indeed revealed that the sensory abilities of an animal are reflected in the amount of neural tissue devoted to the processing of the dominant sensory cues which drive its behavior (Gronenberg and Hölldobler, 1999; Stöckl et al., 2016a; Immonen et al., 2017). For instance, nocturnal hawkmoths invest more in olfactory brain areas in comparison to diurnal hawkmoths, which invest more heavily in visual processing (Stöckl et al., 2016a). Such tradeoffs between enlarging important brain regions at the expense of regions not needed to the same degree are found even in higher order brain areas as long as they could be linked to processing information from a single sensory modality (Gronenberg and Hölldobler, 1999; Gronenberg et al., 2008; Stöckl et al., 2016a). Besides the finding that differences in neuropil volumes can indicate innate functional differences between species, volume changes due to plasticity within species can also hint at underlying functions. For instance, foraging bees have significantly larger mushroom bodies compared to non-foraging nursing bees of the same age (Farris et al., 2001; Fahrbach, 2006; Riveros and Gronenberg, 2010), a change that in honeybees can be attributed to the greater demands imposed on long-term memory while foraging in a rich visual environment (Gronenberg and Couvillon, 2010). This finding was confirmed in butterflies, where wild-caught individuals possessed a massively enlarged mushroom body compared to individuals raised in captivity (Montgomery et al., 2016). Recently, the overall volumetric changes in honeybee mushroom bodies were linked to distinct changes in the fine-structure of specific synapses (Groh et al., 2012), suggesting direct functional relevance for memory processes. Whereas not all small-scale structural changes translate into larger volume differences (Hourcade et al., 2010), this example shows that volumetric analysis of brain areas can indeed deliver a meaningful starting point for highlighting regions of interest for closer examination. Although such links have been revealed in sensory information processing and memory circuits, it has remained unclear whether similar effects can be found with respect to behavioral control mechanisms, e.g., navigational strategies. Are specific, elaborate navigation behaviors reflected in the structure of the brain regions that control them?

To address this question, we have investigated the brains of two species of closely related, nocturnal moths, the Australian Bogong moth (*Agrotis infusa*) and the Turnip moth (*A. segetum*). The Bogong moth is a long-distance migrant (Heinze and Warrant, 2016; Warrant et al., 2016), while the Turnip moth is an opportunistic, agricultural pest species without clear seasonal migrations (Esbjerg and Sigsgaard, 2014). Although short distance seasonal movements (40–60 km) matching prevailing winds have been reported for this species in China (Guo et al., 2015), Turnip moths do not show reproductive diapause, a hallmark of most truly migratory insects (oogenesis-flight syndrome; Dingle, 1972; Zhan et al., 2011; Guo et al., 2015). In contrast, the Bogong moth’s migrations are reminiscent of the famous Monarch butterfly, a species that performs spectacular yearly migrations across North America, albeit during the day (Merlin et al., 2012; Guerra and Reppert, 2015; Reppert et al., 2016). Each spring an estimated 2 billion moths migrate over 1000 km from their breeding grounds in various regions of southeast Australia to the alpine regions of the Australian Alps, where they locate specific caves for spending the summer (Warrant et al., 2016). In the cool and constant climate of these alpine caves they enter a dormant state (called aestivation) for 3–4 months, after which, at the beginning of the autumn, they carry out the long return trip to their breeding grounds to mate, reproduce and die (Warrant et al., 2016). Unlike diurnal migrants, these moths cannot use the sun and other sun-derived sky-compass cues during their nocturnal migratory flights, but instead rely on an unknown combination of nocturnal visual and, possibly, magnetic-field based compass cues (Heinze and Warrant, 2016; Warrant et al., 2016).

The regions of the insect brain that have been generally implicated in processing compass stimuli and controlling migratory behavior have collectively been called the “compass neuropils” in the Monarch butterfly (Heinze et al., 2013) and comprise the central complex (CX), the lateral complex (LX) and the anterior optic tubercles (AOTU; Heinze and Reppert, 2012; Heinze et al., 2013; Pfeiffer and Homberg, 2014). These regions are highly conserved across all insects (Homberg, 2008; Ito et al., 2014; Immonen et al., 2017) and likely play a major role in all orientation behaviors, carrying out multiple computational steps from sensory integration to generation of premotor commands (Heinze and Homberg, 2007; Seelig and Jayaraman, 2013, 2015; Martin et al., 2015; Namiki and Kanzaki, 2016a, b).

In the work presented here, we generated an average-shape atlas of these regions and used volumetric analysis to identify potential differences in their layout between the migratory and non-migratory moths. We further compared the results to identical data from Monarch butterflies (Heinze et al., 2013). Whereas we found differences in the size of the nodular unit of the AOTU, the PB, as well as in parts of the LX between the two moths, these did not reflect migratory behavior across all three species. We conclude that the phylogenetic relationship is clearly the biggest predictor of brain anatomy and that navigational strategy, at least in the case of long-distance migration in lepidopteran insects, is not easily deductible from anatomical features at the level of neuropils.

## Materials and Methods

### Animals

Australian Bogong moths (*Agrotis infusa*) were collected either in early January or in late October (2013–2015) from caves near the peak of South Ramshead mountain, in Kosciuszko National Park, NSW, Australia. The moths had already undergone their forward spring migration and were in an aestivating state when captured. They were brought to Lund, Sweden and kept in the aestivating state in an artificial cave environment, set to temperatures of 6°C at night and 10°C during the day under long-day conditions (16 h, dim illumination:8 h, dark). Diluted honey solution (10 g honey, 10 g sucrose, in 1 l of water) was provided as food *ad libitum*. The animals used were dissected within 5 months of capture.

Turnip moths (*Agrotis segetum*) were bred in captivity at Lund University at 21°C (13 h, light:11 h, dark). The moths used were from populations from the years 2013 to 2016. Twelve moths of each species were used for reconstruction and standardization. Both female and male individuals of Bogong moths were used, while for Turnip moths we used only male individuals.

Raw data from Monarch butterflies was published by Heinze et al. (2013) and was reanalyzed in the current article to enable direct comparisons to the moth species.

### Immunocytochemistry

The moth brains were dissected out of the head capsule in fixative (1% formaldehyde/zinc-chloride in Hepes-buffered saline (HBS; Ott, 2008)) and fixed overnight at 4°C. The brains were then subjected to rinses (8 × 20 min) in HBS, during which tracheae and the retinae were removed. The Bogong moth brains were then bleached with 10% H_2_O_2_ in Tris/HCl buffer for 6 h (Stöckl and Heinze, 2015) while *A. segetum* brains were bleached in 1% H_2_O_2_ in Tris/HCl buffer, exchanged every hour for 6 h. Following a wash in Tris/HCl buffer (3 × 10 min) the brains were incubated in a fresh mixture of methanol and dimethylsulfoxide solution (DMSO, 80:20; Bogong moths: 70 min, Turnip moths: 85 min; Ott, 2008). After an additional Tris/HCl buffer wash (3 × 10 min) the brains were pre-incubated with 5% normal goat serum (NGS) in 0.01 M phosphate-buffered saline (PBS), containing 0.3% TritonX-100 (PBT), overnight at 4°C. For visualization of neuropils, they were subsequently incubated with mouse derived primary antibodies against the presynaptic vesicle protein synapsin (Klagges et al., 1996; 1:25 in 0.01 M PBT containing 1% NGS for 5–6 days in 4°C). Following extensive washing in PBT (8 × 20 min) the brains were incubated with Cy5-conjugated secondary antibody (goat anti-mouse; 1:300 in 0.01 M PBT with 1% NGS for 5 days in 4°C). After rinsing in 0.01 M PBT (6 × 30 min) and 0.1 M PBS (2 × 30 min) the samples were dehydrated with an ascending ethanol series (50%, 70%, 90%, 95%, 2 × 100%, 15 min each). Thereafter, the preparations were transferred to a fresh mixture of methyl salicylate and ethanol (1:1) for 15 min, followed by pure methyl salicylate for a minimum of 60 min. Finally, the brains were embedded in Permount between two coverslips, using a stack of plastic spacers to avoid compression.

### Intracellular Dye Injections

Neurons were injected with neurobiotin in the context of intracellular recordings (performed according to standard methods, for details see e.g., Heinze and Reppert, 2011). We used glass microelectrodes of 50–150 MΩ resistance that were filled with 4% Neurobiotin solution (in 1 M KCl), backed up with 1 M KCl. After impaling a cell, a positive current (1–3 nA, for 1–3 min) was applied to the electrode in order to iontophoretically eject neurobiotin molecules from the electrode tip. The brain was dissected out of the head capsule, and fixed in neurobiotin fixative (4% paraformaldehyde, 0.25% glutaraldehyde, 2% saturated picric acid, in 0.01 M PBS) overnight at 4°C. Brains were then rinsed 4 × 15 min with 0.1 M PBS and incubated with Cy3-conjugated streptavidin (1:1000, in 0.01 M PBT) for 3 days at 4°C. Brains were then washed 4 × 20 min in PBT and 2 × 20 min in PBS, after which they were dehydrated in an ethanol series of increasing concentrations, cleared in methyl salicylate, and mounted between two coverslips using Permount (details identical as for immunohistochemistry).

### Image Acquisition

We imaged the labeled samples using a confocal laser scanning microscope (Zeiss LSM 510) equipped with a 25× long distance objective (LD LCI Plan-Apochromat 25×/0.8 Imm Corr DIC; Zeiss) with either the 633 nm laser line (Cy5-labels) or with the 561 nm laser line (Cy3-labels). To cover the entire region of interest, 2–3 contiguous image-stacks had to be acquired per brain. To minimize photo-bleaching and to maximize scanning efficiency, anti-synapsin-labeled samples were imaged at a resolution close to the final desired voxel-size of 1 × 1 × 1 μm: 512 × 512 pixels per stack in *x-y* direction (voxel-size: 0.99 × 0.99 μm) and 1.03 μm in *z* direction, using bidirectional scanning. Injected neurons were imaged at a voxel size of 0.29 × 0.29 × 0.89 μm using the same objective.

### Image Processing and 3D Reconstruction

The image stacks for each sample were aligned, merged and resampled to a voxel size of 1 × 1 × 1 μm using the software Fiji or Amira 5.3. These image data were then used as raw data for semi-manual image segmentation. Hereby we first created a label field, in Amira 5.3, in which voxels were assigned a neuropil identity. Neuropil boundaries of key optical sections were labeled in every spatial plane (*x-y, x-z, y-z*), generating a scaffold for each neuropil of interest. The scaffolds were automatically completed to contain all voxels that belong to each neuropil by the “wrap” function in Amira. Finally, a triangulated surface model was generated from the segmented label-fields. The neuropils included were upper and lower divisions of the central body (CBU, CBL), the noduli (NO), the protocerebral bridge (PB), the lateral accessory lobe (LAL), the gall (GA), the bulb (BU), the upper unit of the AOTU (AOTU-UU), the lower unit of the AOTU (AOTU-LU) and the nodular unit of the AOTU (AOTU-NU). The color code introduced for these neuropils by Heinze et al. (2013) was used as a template.

Neurons were traced manually in 3D using the skeletonize plug-in for Amira 5.3 (Schmitt et al., 2004; Evers et al., 2005). First, confocal image stacks containing a labeled neuron were aligned into a common frame of reference. Second, the skeleton of the neuron was traced by manually selecting key points along the neuron’s path as well as selecting all branch points. The resulting straight neuron segments were fitted to the brightness information of the image stack to obtain realistic midline curvature and diameter for each branch.

### Standardization

For standardization we reconstructed the neuropils of interest from twelve individuals for each species. We chose the computational morphometry toolkit (CMTK) as standardization method, implemented by the iterative shape averaging (ISA) protocol (Rohlfing et al., 2001; el Jundi and Heinze, in press), given that this method has been used successfully for many species, including the “compass neuropils” of the Monarch butterfly (Brandt et al., 2005; Kurylas et al., 2008; Kvello et al., 2009; Wei et al., 2010). In the ISA protocol gray values are used as the basis for image comparison. As the compass neuropils were scanned from the center of the brain, the edges of the individual image stacks visible in the merged overall image stack contained the highest contrast, however did not correspond to any brain structure. These boundaries thus had to be eliminated to prevent the algorithm to align the boundaries of the image stacks, rather than the internal brain structures. Following the method introduced by el Jundi et al. (2009a) and Heinze et al. (2013), we therefore removed all image information located further than 25 voxels away from the labeled neuropil boundaries. This generated a “cut-out” image stack with consistent outer borders. These image stacks provided the raw material for the ISA protocol. Before the actual registration process, a reference brain was chosen, which in terms of shape and volume represented the population average of the 12 reconstructed individual brains most closely. This is crucial since the reference brain strongly influences the volume of the final result of the ISA protocol. In general terms, the ISA protocol is a two-step procedure, in which an affine registration is followed by an elastic registration, which is iterated multiple times. In the first step, the reference brain is used as a template to which all remaining brains are aligned through affine registration. The affine registration was carried out twice, first with 6 degrees of freedom (rotation and translation along the three cardinal axes), then with 9 degrees of freedom, i.e., additional scaling along all three cardinal axes. This initial procedure compensated for difference in rotation, size and position between the individual image stacks. The registered brains were then averaged and the resulting coarse average brain was used as the reference for the elastic registration. This process adjusts shape differences between brains by introducing local deformations to maximize image similarity. This process is repeated five times, each round using the previously generated averaged image stack as the new template. This leads to the final average image stack. The set of registration parameters obtained from each individual brain were then applied to the label field data from the same brain, yielding a set of 12 registered label fields. Using the shape based averaging method (Rohlfing and Maurer, 2007), a standardized surface reconstruction was calculated. All computations required to run the ISA protocol were performed on the MaRC2 HPC Linux cluster based at the IT-facilities of the University of Marburg, Germany. Using 64 cores (AMD 6276 at 2.3 GHz) and approximately 4 GB of shared memory, the ISA protocol took approximately 5 days to complete. The atlases are available for download as well as for interactive use at the InsectBrainDatabase (Bogong moth: https://www.insectbraindb.org/species/2/; Turnip moth: www.insectbraindb.org/species/21/).

### Volumetric Analysis

For each reconstructed brain, we extracted volume information for each neuropil from label-field data by using the *material statistics* tool in Amira 5.3. All raw data is available in Supplementary Tables S1–S3. We calculated relative volumes by normalization, i.e., the absolute volumes were divided by the total volume of all neuropils of each brain, thus eliminating effects of size differences between individuals and species. To assess the overall investment into each type of neuropil, we summed the values of the right and left hemispheres within each brain.

The comparison of relative volumes between the neuropils of the two moth species was carried out by the Mann-Whitney U test, given that the volume distribution of some neuropils deviated significantly from normal (tested with Shapiro-Wilk normality test). The means and standard deviations of each neuropil were calculated and displayed. For three species comparison between the moths and the Monarch butterfly, we used non-parametric ANOVA (Kruskal-Wallis test) with Dunn’s test for multiple comparisons. As the gall-region of the LX was not segmented separately in the Monarch butterfly, and the strap-region of the AOTU was not found in the two moths, we combined the gall with the LAL in the moth species and included the strap with the AOTU-lower unit in the Monarch butterfly for the three-species comparison. All the statistical analyses above were performed in Graphpad-Prism 6.0 software.

To investigate whether differences in neuropil volume resulted from true differences in the size of the neuropils of interest (i.e., grade shifts), rather than apparent differences in relative size caused by non-isometric scaling, we carried out standardized major axis regression analysis on all neuropils (Warton et al., 2006; Ott and Rogers, 2010), first between the two moth species and second between all three species. We used the SMATR v.3 package for R, as described by Warton et al. (2012). This method assumes an allometric relationship of the form *y* = *a* * *x^b^*, which translates to the linear relationship log(*y*) = log(*x*) * *b* + log(*a*). In cases where we did not find differences in allometric scaling between species (equal slopes *b*), we could test for differences in the *y*-axis intercept, or elevation (log(*a*)), called a grade shift. All neuropils examined fulfilled this criterion. If grade shifts exist, they indicate a true difference in neuropil volume across species (Ott and Rogers, 2010). The extent of the shift in elevation was quantified as the grade shift index (GSI) as described by Ott and Rogers (2010). Additionally we tested if the scaling relationship of a neuropil (using the common slope of the examined species) was different from isometric scaling (i.e., the neuropil scales at the same rate as the overall neuropils). The difference in slope from isometric scaling is defined as the slope index (SI).

### Neuron Registration

Neurons reconstructed from individual brains were mapped into the standard atlas following the method described in detail in el Jundi et al. (2009a), applied according to Heinze et al. (2013). In short, we first reconstructed the neuropils innervated by the neuron of interest based on the background fluorescence in the image stack containing the neuron. These were then affinely and elastically registered onto the standard atlas. The resulting transformation parameters were then applied to the neuron reconstruction itself and yielded a neuron that was matched to the reference frame of the standard atlas, as well as locally adjusted in shape to compensate for any distortions present in the individual brain.

Naming of neuropils follows the naming scheme developed by the Brain Name Working Group (Ito et al., 2014) and differ in some neuropils from the names used by Heinze et al. (2013) in the Monarch butterfly. All neuropil orientations are stated according to body axis (not neuraxis).

## Results

### Proposed Migration-Relevant Neuropils in Noctuid Moths

**Figure 1:**
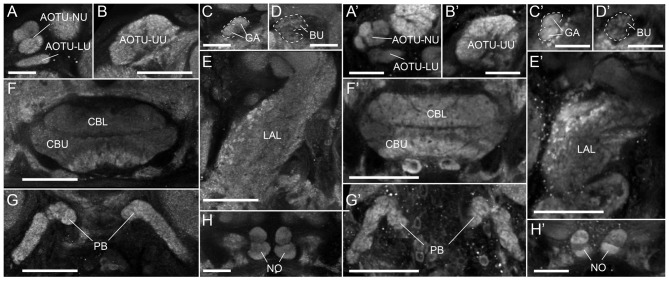
The anatomy of proposed navigation-relevant neuropils in two species of noctuid moths. **(A–H)** Single confocal sections (frontal orientation) of an anti-synapsin labeled Bogong moth brain, scanned directly from whole-mount preparation. **(A)** Lower unit complex (LUC) of the anterior optic tubercle (AOTU) consisting of lower unit (LU) and nodular unit (NU). **(B)** Upper unit of the AOTU (UU). **(C)** The gall (GA) of the lateral complex (LX). **(D)** The bulb (BU) of the LX. **(E)** The lateral accessory lobe (LAL) or the LX. **(F)** The upper and lower divisions of the central body (CBU, CBL) of the central complex (CX). **(G)** The protocerebral bridge (PB) of the CX. **(H)** The noduli (NO) of the CX. **(A′–H′)** As **(A–H)**, but for the Turnip moth. **(G’)** is a maximal intensity projection of three individual images. Scale bars: **(A,C,D,H)** 50 μm; **(B,E,F,G)** 100 μm.

**Figure 2:**
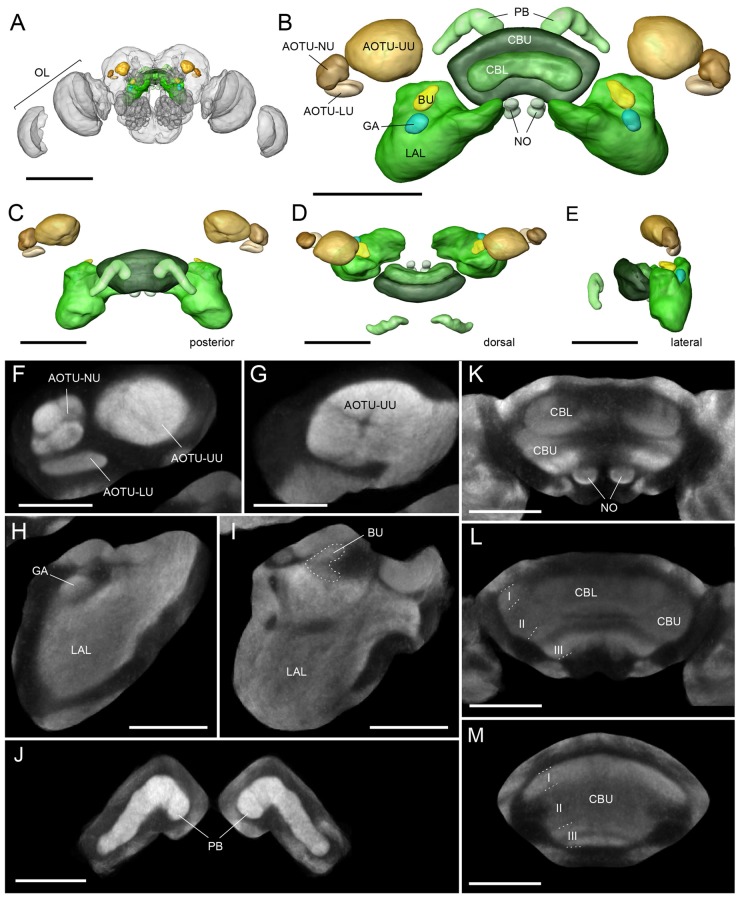
The standardized average-shape Bogong moth “compass neuropils”. **(A)** Anterior view of the Bogong moth brain with regions of interest highlighted in color. **(B)** Anterior view of the finalized standard atlas. Shown are surface renderings of average-shape label-fields. **(C–E)** As for **(A)** but posterior view **(C)** dorsal view **(D)**, and lateral view **(E)**. **(F–L)** Single optical sections of the averaged confocal data stack resulting from the standardization protocol. **(F)** Lower unit complex of the AOTU with lower unit (LU) and nodular unit (NU), shown with upper unit of the AOTU (AOTU-UU). **(G)** More posterior level of the AOTU-UU. **(H,I)** Different levels of the lateral complex showing the lateral accessory lobes (LAL), gall (GA) and bulb (BU). The BU is outlined with a dashed line. **(J–M)** Different levels of the central complex showing the lower division of the central body (CBL), upper division of the central body (CBU), noduli (NO) and the protocerebral bridge (PB). Scale bars: **(A)** 500 μm; **(B–D)** 200 μm; **(F,G)** 80 μm; **(J–M)** 100 μm. OL, optic lobe.

The Bogong and Turnip moth’s counterparts of the Monarch butterfly’s “compass neuropils” also consist of the four compartments of the CX, the three compartments of the LX, and one large and several small compartments of the AOTU (Figures 1, 2). The CX can be divided into the upper and lower division of the central body (CBU and CBL), the paired NO, and the posteriorly located PB. The overall shape of these neuropils resembles that of other lepidopteran insects: the PB is discontinuous across the midline, the CBL has an elongated, straight shape, located anteriorly of the much larger CBU, and the NO are small, ventrally located structures consisting of two major subunits, one large and one small (Figures 1, 2). Whereas horizontal layers are clearly visible in the CBU (three major layers from dorsal to ventral; Figures 1F,F′, 2K–M), the columnar neuroarchitecture typical for the insect CX is not pronounced on the level of neuropils in the Bogong moth.

The LX are located anterior-ventrally on either side of the CX and consist of the large LAL (Figures 1E,E′) and two small neuropils, the bulb (BU; Figures 1D,D′) and the gall (GA; Figures 1C,C′). In the Monarch butterfly these small regions had been originally named the lateral triangle and the anterior loblet (Heinze and Reppert, 2012). Typical for the LAL, the boundaries of this region are highly defined only on its anterior and medial side. Dorsally, ventrally and laterally it merges with the surrounding neuropil regions. These boundaries have hence been defined in accordance with criteria used in the Monarch butterfly, *Drosophila* and the dung-beetle. The gall on the other hand is clearly visible as a small structure on the anterior face of the LAL, just posterior of the antennal lobe. It consists of two fused ellipsoids of smooth, even appearance in synapsin-labeled preparations (Figures 1C,C′). Immediately dorsal of the gall lies the bulb. This neuropil consists of many small microglomeruli (Figures 1D,D′) and, due to their irregular spatial arrangement, the overall neuropil shape and volume are comparably variable across individuals. The bulbs are nevertheless well-defined by providing a cap-like end to the isthmus tracts leaving the CBL on either side, while laterally and dorsally bordering the mushroom body lobes (Figures 1D,D′).

The AOTU consists of the large upper unit (AOTU-UU) as well as the much smaller lower unit complex (LUC), which can be divided into the lower unit (AOTU-LU) and the nodular unit (AOTU-NU; Figures 1A,A′, 2F). The latter can be further divided into four glomerular sub-compartments. While the small subunits are highly defined and can be easily separated from other brain regions, the upper unit merges medially with the surrounding superior protocerebrum, but can nevertheless be distinguished by its brighter synapsin labeling (Figures 1B,B′, 2G).

### Average-Shape Atlases of Migration-Relevant Neuropils

**Figure 3:**
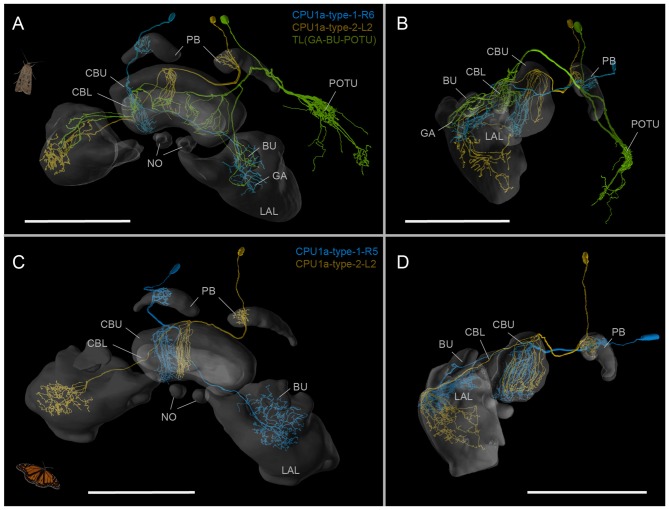
The Bogong moth standard atlas as reference frame for neuron morphologies. **(A,B)** Three intracellularly injected and reconstructed neurons from the CX, mapped into the standard atlas by elastic registration. The TL-(GA-BU-POTU)-neuron is reported here for the first time. Oblique frontal view **(A)**; lateral view **(B)**. **(C,D)** Neuron of the same types as the cells with identical color in **(A)** (two types of CPU1a-neurons), but from the Monarch butterfly, registered into the standard atlas of the Monarch butterfly compass neuropils. Note the high degree of similarity between the species. Oblique frontal view **(C)**; lateral view **(D)**. Data from Heinze et al. (2013). Scale bars: 200 μm; Abbreviations: CBL, lower division of the central body; CBU, upper division of the central body; BU, bulb; LAL, lateral accessory lobe; GA, gall; PB, protocerebral bridge; NO, noduli; POTU, posterior optic tubercle. Images obtained at www.insectbraindb.org.

To generate a baseline for future anatomical work on the neural circuits underlying the Bogong moth’s migratory behavior, we have used the ISA protocol (ISA), implemented through the CMTK toolkit, to generate a standardized, shape averaged version of the Bogong and Turnip moth’s counterparts of the Monarch butterfly “compass neuropils” (Figure 2). These standards are based on 12 individual brains each and now provide a reference atlas for registration of anatomical data from any individual of both species. At a voxel-size of 1 × 1 × 1 μm, the resolution of this atlas is equivalent to that of the Monarch butterfly and exceeds that of all other species in which brain atlases have been published, with the exception of *Drosophila*. To illustrate the functionality as standardized Bogong moth reference neuropils, we have registered three intracellularly filled neurons into this standard atlas, generating a starting point for collecting an increasing amount of anatomical data (Figure 3). These neurons included two columnar neurons of the CX (CPU1-neurons), which are well described in other insects (e.g., Monarch butterfly; Figures 3C,D; Heinze et al., 2013; locusts, el Jundi et al., 2009a), as well as a type of CX neuron described here for the first time. This cell (TL-(GA-BU-POTU)) innervates the CBL, the gall, the bulbs and the posterior optic tubercle (the latter is not part of the standard atlas due to its high variability in size, shape and location; Figures 3A,B), i.e., most compass-related regions of the insect CX. This broad innervation pattern combined with an unpronounced anatomical polarity (no clear input and output regions based on morphological criteria) suggests a modulatory role for this neuron within the compass circuit.

**Figure 4:**
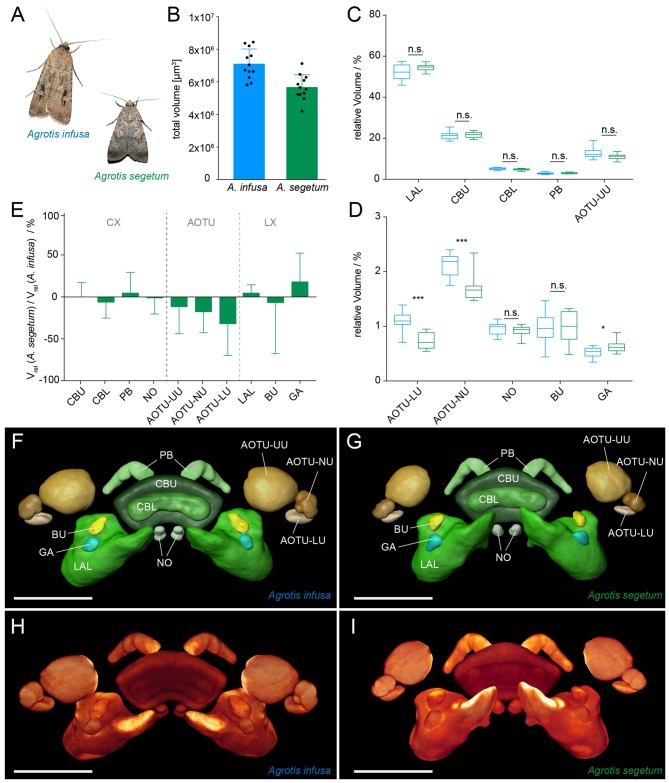
Comparison of migration-relevant neuropils between the migratory Bogong moth (*Agrotis infusa*) and the non-migratory Turnip moth (*A. segetum*). **(A)** Photographs of both species (Bogong moth photo courtesy of Ajay Narendra); wingspan: 40–50 mm (*A. infusa*), 32–42 mm (*A. segetum*). **(B)** Total volume of the combined compass neuropils of both species. Individual data points are shown together with mean and standard deviation. **(C,D)** Box plots of relative volumes of each examined neuropil (normalized to the total volume); whiskers: data range; box: 25% and 75% percentiles; line: median. Bogong moth, blue; Turnip moth, green. **(C)** Large neuropils; **(D)** small neuropils. Asterisks indicate significance levels resulting from Mann-Whitney U test. **(E)** Ratio of neuropil volumes between Turnip moth and Bogong moth. Values smaller than one indicate larger volumes in the Bogong moth, while values larger than one indicate larger volumes in the Turnip moth. Error bars are summed relative standard deviations of corresponding neuropils from both species. **(F,G)** Surface rendering of standardized label-fields of average-shape atlases of the Bogong moth **(F)** and the Turnip moth **(G)**. **(H,I)** Direct volume rendering of image stack resulting from the standardization protocol. **(H)** Bogong moth; **(I)** Turnip moth. Scale bars: 200 μm. Abbreviations: AOTU, anterior optic tubercle; UU, upper unit; LU, lower unit; NU, nodular unit; CBL, lower division of the central body; CBU, upper division of the central body; BU, bulb; LAL, lateral accessory lobe; GA, gall; PB protocerebral bridge; NO, noduli.

The same 12 randomly selected brains also provide a representative sample of neuropil volumes for quantitative analysis (Figures 4B–D). Both absolute and relative volumes (fractions of the overall volume of all compass neuropils) were calculated to serve as a basis for quantitative, interspecies comparisons. So as not to overestimate the relative investment into unpaired neuropils (CBU and CBL), we summed the right and left hemispheres of all paired brain areas for all volume calculations, similar to previous work (Tables 1, 2). In the following we compare the neuropil volumes of the Bogong moth to the Turnip moth and to reanalyzed, previously published data from the diurnal migratory Monarch butterfly (Heinze et al., 2013).

**Table 1:** Volumes of neuropils of Bogong moth (*Agrotis infusa*).

	Mean absolute volume/μm^3^	SD/± μm^3^	Mean relative volume/%	Relative SD/±%
CBU	1.51 × 10^6^	1.71 × 10^5^	21.43	10.0
CBL	3.59 × 10^5^	4.59 × 10^4^	5.08	9.4
PB	2.03 × 10^5^	4.00 × 10^4^	2.85	13.3
NO	6.80 × 10^4^	1.33 × 10^4^	0.96	12.0
LAL	3.71 × 10^6^	5.39 × 10^5^	52.25	6.9
BU	7.06 × 10^4^	2.58 × 10^4^	0.99	30.4
GA	3.78 × 10^4^	9.10 × 10^3^	0.53	17.6
AOTU-UU	9.07 × 10^5^	2.60 × 10^5^	12.69	19.1
AOTU-NU	1.51 × 10^5^	2.49 × 10^4^	2.12	10.0
AOTU-LU	7.88 × 10^4^	2.15 × 10^4^	1.10	17.0

**Table 2:** Volumes of neuropils of Turnip moth (*Agrotis segetum*).

	Mean absolute volume/μm^3^	SD/± μm^3^	Mean relative volume/%	Relative SD/±%
CBU	1.16 × 10^6^	1.52 × 10^5^	21.05	6.13
CBL	2.57 × 10^5^	4.94 × 10^4^	4.65	13.12
PB	1.65 × 10^5^	3.46 × 10^4^	2.99	14.06
NO	5.27 × 10^4^	4.24 × 10^3^	0.96	4.85
LAL	2.98 × 10^6^	2.65 × 10^5^	54.30	4.52
BU	5.48 × 10^4^	1.04 × 10^4^	1.01	24.10
GA	3.33 × 10^4^	3.78 × 10^3^	0.61	14.16
AOTU-UU	6.55 × 10^5^	4.93 × 10^4^	11.97	5.98
AOTU-NU	9.69 × 10^4^	1.04 × 10^4^	1.77	10.87
AOTU-LU	3.79 × 10^4^	1.14 × 10^4^	0.69	25.70

### Comparison between Bogong Moth and Turnip Moth

All neuropils found in the Bogong moth were also identified in the brain of the Turnip moth. Moreover, the overall shape of all regions resembled that of the Bogong moth closely and no principal differences were obvious despite the difference in behavioral strategy (Figures 4F–I). The total absolute size of the Turnip moth’s combined compass neuropils was approximately 20% smaller than in the Bogong moth, in line with its smaller body size (Figure 4B; *p* < 0.001; unpaired, two-tailed *t-test)*. To quantitatively compare individual neuropils of the two species, we performed two types of analyses. First, we compared relative volumes of all neuropils, and second, we carried out standardized major axis regression analysis. The first method has previous been used in many species and is thus aimed at providing a basis for direct comparison of the presented data with those studies. The second analysis yields a more robust estimate of true difference between species, as it does not assume isometric scaling of all included neuropils. It is therefore a more rigorous basis for drawing functional conclusions.

When normalized to overall size, the relative volumes between the two species matched remarkably well for all parts of the CX (no significant differences, Mann-Whitney U test). The data for the LX also revealed no differences for the LAL and the BU, while showing a small, but weakly significant (*p* = 0.039) volume increase in the Turnip moth’s gall region. However, in contrast, all neuropils of the AOTU showed a consistent trend towards smaller size in the Turnip moth (between 7%–25%). Of these, only the small subunits (lower and nodular unit) were significantly smaller (*p* < 0.001), whereas the upper unit just missed significance (*p* = 0.068; Figures 4C–E).

**Figure 5:**
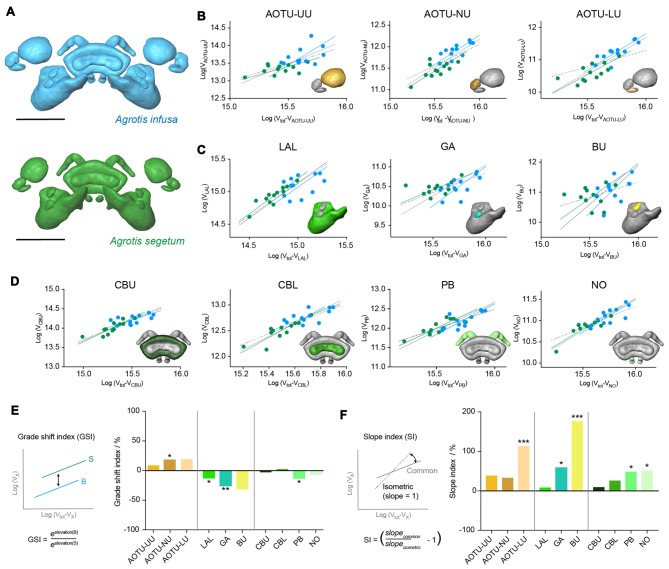
Results from standardized major axis regression analysis comparing the Bogong moth and the Turnip moth compass neuropils. **(A)** Surface rendering of neuropils in each species. **(B–D)** Regression analysis data; colors matching neuropil color in **(A)**. For each neuropil individual data-points are shown together with the individual regression line of each species, the best-fit common regression line (gray) and the isometric expectation (dotted). The inset depicts the respective neuropil for each graph highlighted in color. **(B)** Neuropils of the AOTU. **(C)** Neuropils of the LX. **(D)** Neuropils of the CX. **(E)** Analysis of grade shift indices (GSI) of all neuropils. Positive values indicate a shift towards bigger volumes in the Bogong moth, while negative values indicate smaller volumes in the Bogong moth. Left: schematic illustration and formula of how the GSI is calculated. Asterisks indicate significance level of GSI. **(F)** Analysis of slope index (SI) for all neuropils. Values larger than zero indicate steeper than isometric scaling. Asterisks illustrate significant deviations from isometric scaling. Left: schematic illustration and formula of how the SI is calculated. Scale bars: 200 μm. Abbreviations: AOTU, anterior optic tubercle; UU, upper unit; LU, lower unit; NU, nodular unit; CBL, lower division of the central body; CBU, upper division of the central body; BU, bulb; LAL, lateral accessory lobe; GA, gall; PB protocerebral bridge; NO, noduli.

To examine whether these differences in relative volume are truly independent of overall size, we analyzed the detailed allometric relationships of all involved neuropils (using standardized major axis regression analysis in R; Figure 5). When plotting the absolute volume of each region against the total remaining volume of the compass neuropils, all regions showed identical slopes between both species (a common slope described the data best). In about half the cases this slope was not significantly different from the expected isometric relationship, in which the neuropil volume would increase at the same rate as the total volume (Figure 5F). Highly significant exceptions were the AOTU-LU and the bulbs, while the PB, NO and the gall showed a weakly significant deviation. In all cases the neuropils showed disproportionately large size increases, i.e., the individual volume increased faster in size than the total volume. This finding is of key importance when interpreting the results of the relative volume analysis, which due to the normalization to overall volume, assumes isometry for all neuropils.

When analyzing the vertical displacement of the regression lines between both species (the grade-shift, expressed as the GSI), the AOTU-NU and the gall yielded significant differences, with the AOTU-NU being larger in the Bogong moth and the gall being larger in the Turnip moth, in line with the simple analysis of mean relative volumes (Figures 5B–E). Moreover, the LAL and the PB also showed a significant GSI towards larger volumes in the Turnip moth, that were obscured in the earlier analysis. Interestingly, the highly significant difference found in the AOTU-LU for the mean volumes was not confirmed in the more detailed analysis. This is because the simple analysis assumes an isometric scaling of the neuropils. Given that the AOTU-LU volume increases faster than expected with larger brains, and the Bogong moth brain is generally larger than the Turnip moth brain, the difference in relative volume is best explained by a shift of the neuropil volumes along the same regression line.

### Comparison to the Migratory Monarch Butterfly

**Figure 6:**
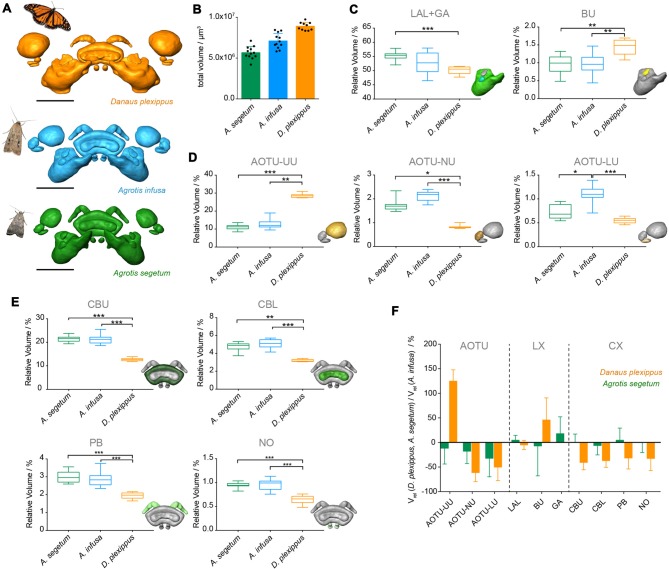
Comparison of compass neuropils between migratory and non-migratory moths (Bogong moth, *A. infusa*; Turnip moth, *A. segetum*) and the migratory Monarch butterfly (*Danaus plexippus*). **(A)** Surface rendering of neuropils in each species (shown in correct relative size), shown with image of each species. **(B)** Total volumes of the combined compass neuropils. Individual data points are shown together with mean and standard deviation. **(C–E)** Box plots of relative volumes of each examined neuropil (normalized to the total volume) whiskers: data range; box: 25% and 75% percentiles; line: median. Bogong moth, blue; Turnip moth, green; Monarch butterfly, orange. The inset depicts the respective neuropil for each graph highlighted in color. Asterisks indicate significance levels resulting from ANOVA analysis (with Dunn’s test for multiple comparisons). **(C)** LX neuropils. **(D)** AOTU neuropils. **(E)** CX neuropils. **(F)** Ratio of neuropil volumes between Turnip moth and Bogong moth (green) and the Monarch butterfly and the Bogong moth. Values smaller than one indicate larger volumes in the Bogong moth, while values larger than one indicate smaller volumes in the Bogong moth. Error bars are summed relative standard deviations of corresponding neuropils from both species. Scale bars: 200 μm. Abbreviations: AOTU, anterior optic tubercle; UU, upper unit; LU, lower unit; NU, nodular unit; CBL, lower division of the central body; CBU, upper division of the central body; BU, bulb; LAL, lateral accessory lobe; GA, gall; PB protocerebral bridge; NO, noduli.

**Figure 7:**
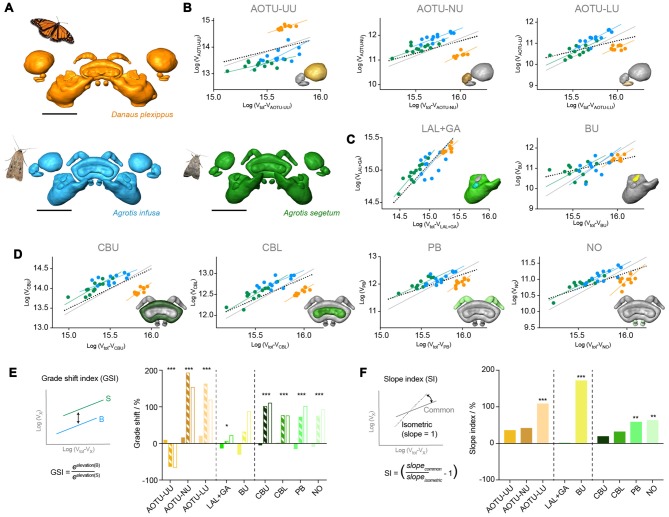
Results from standardized major axis regression analysis comparing the Bogong moth, the Turnip moth and the Monarch butterfly compass neuropils. **(A)** Surface rendering of neuropils in each species (shown in correct relative size), shown with image of each species. **(B–D)** Regression analysis data; colors matching neuropil color in **(A)**. For each neuropil individual data-points are shown together with each species’ individual regression line, the best-fit common regression line (gray) and the isometric expectation (dotted). The inset depicts the respective neuropil for each graph highlighted in color. **(B)** Neuropils of the AOTU. **(C)** Neuropils of the LX. **(D)** Neuropils of the CX. **(E)** Analysis of GSI of all neuropils. Solid bars: Bogong moth—Turnip moth comparison (values > 0: Bogong moth bigger; values < 0: Bogong moth smaller); hatched bars: Bogong moth—Monarch butterfly comparison (values > 0: Bogong moth bigger; values < 0: Bogong moth smaller); open bars: Turnip moth—Monarch butterfly comparison (values > 0: Turnip moth bigger; values < 0: Turnip moth smaller). Left: schematic illustration and formula of how the GSI is calculated. Asterisks indicate significance level of GSI. **(F)** Analysis of SI for all neuropils. Values larger than zero indicate steeper than isometric scaling. Asterisks illustrate significance level of deviations from isometric scaling. Left: schematic illustration and formula of how the SI is calculated. Scale bars: 200 μm. Abbreviations: AOTU, anterior optic tubercle; UU, upper unit; LU, lower unit; NU, nodular unit; CBL, lower division of the central body; CBU, upper division of the central body; BU, bulb; LAL, lateral accessory lobe; GA, gall; PB protocerebral bridge; NO, noduli.

If the differences found between the two moth species are required for a migratory lifestyle, they should also be reflected in migratory species that are only distantly related to the two moths. To address this hypothesis, we compared our data to previously published data on the migratory Monarch butterfly (Heinze et al., 2013), which had been generated using identical methods (Figures 6, 7). Given that the overall size of the Monarch butterfly is greater than the Bogong moth, the larger total volume of the neuropils (*ca.* 20%) is not unexpected (Figures 6A,B). After normalizing each individual neuropil to the total volume of all combined regions, we compared these relative volumes across all three species (non parametric ANOVA (Kruskal-Wallis test, with Dunn’s test for multiple comparisons)). As the gall region of the LX had not been reconstructed separately from the LAL in the Monarch butterfly, we needed to combine the LAL and the gall in all three species to be able to carry out direct comparisons. Similarly, the strap region of the Monarch butterfly AOTU had to be combined with the AOTU-LU, as the strap does not exist in moths. In summary, whereas neuropils between the two moth species showed only one significant difference in the ANOVA analysis comparing all three species (AOTU-LU), all neuropils of the Monarch butterfly were significantly different from at least one of the two moths. In seven out of nine cases, the Monarch butterfly neuropils were significantly different in volume from both moths (Figures 6C–F). When compared to the Bogong moth, all components of the CX as well as the small subunits of the AOTU were smaller in the Monarch butterfly, whereas the upper unit of the AOTU and the bulb of the LX were significantly larger. The differences were generally more pronounced compared to the differences between the Bogong moth and the Turnip moth, with e.g., the upper unit of the AOTU being more than twice the relative size in the Monarch butterfly and all compartments of the CX being 50% smaller (Figure 6F).

More detailed analyses using standardized major axis regression analysis largely confirmed that differences were most pronounced between the Monarch butterfly and either moth species and were comparably modest between the two moths (Figure 7). All neuropils followed a consistent slope for linear regression analysis, which was significantly different from the expected isometric relationship in the same cases as when comparing only both moth species. This indicates that the steeper than expected scaling of the AOTU-LU, bulbs, PB and NO is a characteristic inherent to those brain areas across the species we investigated (Figure 7F). Highly significant grade shifts were found for all neuropils of the CX and the AOTU (*p* < 0.001), while in the LX only the LAL (plus gall) showed a weakly significant volume decrease in the Monarch butterfly (*p* = 0.013). Consistent with simple comparisons of relative volumes, the GSI indicated larger volumes in the Monarch butterfly for the upper unit of the AOTU, and smaller volumes for all other significantly different neuropils (Figure 7E).

## Discussion

In this article we have examined the effects of navigational strategy on the morphology of central brain neuropils implicated in navigation across three lepidopteran insects. We used two closely related moths, the migratory Bogong moth and the non-migratory Turnip moth, and compared our results to the migratory Monarch butterfly by reanalyzing previously published data from Heinze et al. (2013). In summary, both qualitative and quantitative analysis revealed that the brain regions we examined are highly conserved in overall shape and relative volumes. Between the two moths, clear differences were found in the most peripheral region, the AOTU, in regions of the LX and in the PB of the CX. Between the moths and the Monarch butterfly, larger differences in relative volumes were observed in nearly all regions, none of which however reflected migratory lifestyle when examined across all three species. This suggests that, at least for lepidopteran insects, long-distance migratory behavior cannot be easily predicted from brain structure alone, despite the defining role of this behavior for a species’ natural history.

### The Compass Neuropils Are Highly Conserved Across Species

When we compared the overall layout of the average-shape neuropils, they struck us as highly similar between all three species. First, all major components of the CX, the LX and the AOTU were identified in both moths and in the Monarch butterfly and, second, their spatial arrangement was largely identical. Neither finding is unexpected, despite the fact that considerable variability regarding the relative positioning of those brain areas exists across insects (Heinze and Homberg, 2008; Heinze and Reppert, 2012; Ito et al., 2014; Immonen et al., 2017). This is because this variability largely underlies constraints during brain development (Wegerhoff and Breidbach, 1992; Huetteroth et al., 2010; Boyan and Reichert, 2011) and all three species examined are lepidopteran insects. Thus the relative positioning of the regions under consideration was expected to reflect the highly similar overall brain morphology.

The CX is involved in a multitude of functions that are relevant across all insects. These include locomotor control (Strauss, 2002; Martin et al., 2015), spatial memory (Ofstad et al., 2011; Kuntz et al., 2017), representation of body orientation (Heinze and Homberg, 2007; Seelig and Jayaraman, 2015; Varga and Ritzmann, 2016), and multisensory integration (Homberg, 1994; Ritzmann et al., 2008; Seelig and Jayaraman, 2015). As these functions are important independent of behavioral strategy, sensory environment, and evolutionary history, the underlying neural pathways and circuits are expected to exist in similar form across all insects. Even though some of the identified functions, e.g., involvement in grasshopper singing behavior (Kunst et al., 2011; Balvantray Bhavsar et al., 2017), might be specific to certain species, the fundamental nature of most CX functions demands a high level of structural conservation, which we confirmed in the current work.

Similarly, the LX serves as a crucial input and output relay to and from the CX and has been implicated in generating premotor control signals (Homberg, 1994; Namiki et al., 2014; Namiki and Kanzaki, 2016b). Both roles suggest that the LX regions are indispensable for any insect, despite the fact that they have received little attention so far. This lack of attention is likely due to the fact that the LX components are comparably difficult to identify due to their diffuse boundaries with neighboring neuropils (Heinze and Homberg, 2008; Heinze and Reppert, 2012; Ito et al., 2014; Immonen et al., 2017).

Finally, the AOTU has also been revealed in all insects examined so far, albeit with considerably more variability between species (Homberg et al., 2003; el Jundi et al., 2009b; Mota et al., 2011; Heinze and Reppert, 2012; Pfeiffer and Kinoshita, 2012; Zeller et al., 2015; Immonen et al., 2017). Specifically, the LUC, i.e., the combination of all small AOTU subunits, varies significantly both in numbers of subunits as well as in shape. AOTU function has been most thoroughly characterized in the context of processing visual compass cues, in particular polarized light, in the desert locust (Pfeiffer et al., 2005; Pfeiffer and Homberg, 2007; el Jundi and Homberg, 2012) and the Monarch butterfly (Heinze and Reppert, 2011). Neurons of the AOTU-LUC feed visual input to the bulbs of the LX via two parallel pathways and provide the basis for head-direction encoding in the CX (Pfeiffer et al., 2005; Pfeiffer and Kinoshita, 2012; Heinze et al., 2013; Held et al., 2016). Recently, in *Drosophila*, a highly similar arrangement has been revealed, both anatomically and functionally, albeit with visual landmark information being encoded rather than skylight compass cues (Seelig and Jayaraman, 2013; Omoto et al., 2017). Therefore, one key role of the AOTU-LUC appears to be to relay and probably preprocess visual information essential to encode body orientation, a function that is fundamental to all oriented behavior, again in principle demanding a high degree of conservation. Nevertheless, the variability in this region suggests that the number of parallel pathways, and relative investment into each of them, depends on the nature of the visual information used by each species, allowing variations of a general scheme. Overall, these functional considerations suggest that differences reflecting the sensory environment (e.g., nocturnal vs. diurnal lifestyle) are likely to be found in the more peripheral AOTU, while adaptations to behavioral demands are more likely to be found in the CX and LX, which are more closely associated with motor control.

### Qualitative Difference between Species

If migratory behavior required large-scale, dedicated specializations of the brain, such features would become apparent by comparing the gross morphology of migratory vs. non migratory species’ brains. Across the three analyzed species any such qualitative differences were scarce. While none were identified between the Bogong moth and the Turnip moth, the Monarch butterfly neuropils, in comparison, showed three unique features setting them apart from their moth counterparts. First, the AOTU-LUC consisted of three rather than two sub-regions, the lower unit, the nodular unit and the strap, with the strap being unique to the Monarch butterfly (Heinze and Reppert, 2012). Second, the shape and intrinsic composition of the nodular unit was different in the moths compared to the Monarch butterfly. Even though the strap has been identified in other species of butterfly (Montgomery and Ott, 2014; Montgomery et al., 2016), its specific functional role remains unknown. The same applies to the nodular unit, which is present in all lepidopteran insects examined (el Jundi et al., 2009b; Heinze and Reppert, 2012; Montgomery and Ott, 2014; Montgomery et al., 2016). In the Monarch butterfly it contains a subset of polarization-sensitive TuLAL1a neurons, demonstrating that the AOTU-NU is at least partly involved in compass information processing (Heinze and Reppert, 2011; Heinze et al., 2013). Third, the LX-gall had a different appearance in the Monarch butterfly. Even though this region was not separately segmented in that species, Heinze and Reppert (2012) showed that the Monarch butterfly gall (then called anterior loblet) is a single, disc-shaped region occupying the anterior-most part of the LX. It is characterized by very brightly stained micro-glomeruli of dense synapsin-ir. In contrast, the gall of the moths we examined had a uniform appearance after synapsin-labeling and consists of two fused, yet distinct parts, a dorsal and a ventral bulb (not segmented separately). This is equivalent to the structure of this area in *Drosophila* (Ito et al., 2014) as well as in dung beetles (Immonen et al., 2017). Nothing is known about the function of this neuropil, other than that it is targeted by CL1 neurons (wedge/E-PG neurons in *Drosophila*), which are key components in the representation of body orientation and encoding of sky compass cues across insects (Heinze and Reppert, 2011; Heinze et al., 2013; Seelig and Jayaraman, 2015; Wolff et al., 2015). This suggests that they constitute part of an output pathway from the internal compass that might play a role in guiding behavior. In summary, all three differences suggest that no fundamentally different brain composition is required to mediate the ability for migratory behavior and the identified features likely reflect phylogenetic differences between moths and butterflies.

### Quantitative Differences between Species

Quantitative differences between neuropils in the three species were much more prominent than qualitative differences and were identified with respect to relative neuropil volumes (fractions of total volume) and grade shifts after major axis regression analysis.

Relative volume analysis has been used in many previous studies across many insect species (e.g., el Jundi et al., 2009b; Wei et al., 2010), which are thus directly comparable to our results. Yet, this analysis assumes isometric scaling of all neuropils, an assumption that is consistently not met by several neuropils examined in the current study. As major axis regression analysis does not make this assumption, the results based on this method are more robust and thus provide the basis for all conclusions of this work. Besides providing continuity to previous anatomical work in insects, we note that including the partly contradictory results of both methods side by side illustrates the need for caution when interpreting volumetric differences between brain regions across insects, both in previous and future studies. This is particularly the case when rigorous major axis regressions cannot be performed, e.g., due to low numbers of available individuals, and when effects are small.

In the current work, quantitative differences between the three species were widespread. Most dramatically, the AOTU upper unit was more than twice as large in the Monarch butterfly. This region is involved in color processing in bees (Mota et al., 2013) and contains a visual input pathway to the LAL, parallel to the sky-compass pathway, in locusts (Pfeiffer et al., 2005) and bees (Mota et al., 2011; Pfeiffer and Kinoshita, 2012). Its large size is characteristic for butterflies (Heinze and Reppert, 2012; Montgomery and Ott, 2014; Montgomery et al., 2016) and, together with the highly developed color-vision ability in these insects, suggests that it is essential for visually guided flower foraging. Consistent with this idea, the more olfactory driven nocturnal moths possess a smaller version of this neuropil. In the remaining regions, all components of the CX and the AOTU-LUC subunits were substantially larger in the moths, suggesting that the relative investment in those areas is higher in the moth species investigated compared to the Monarch butterfly. This effect was consistent even when the large AOTU upper unit was excluded from the analysis (Supplementary Figure S1) and does thus not merely result from a distorting effect of this unusually large region in the Monarch butterfly. However, it remains unclear whether the “compass neuropils” as a functional unit might occupy a different proportion of the central brain in moths vs. butterflies.

Between the two moth species, only two quantitative differences were consistently found: the nodular unit of the AOTU was larger in the Bogong moth, while the gall of the LX was smaller in the Bogong moth. A similar difference was identified for parts of the AOTU between two hawkmoth species, one diurnal and one nocturnal (Stöckl et al., 2016a). Here, the AOTU-LU was larger in the nocturnal species, while the upper unit was larger in the diurnal species. Similarly, comparison of individual brains of a diurnal and nocturnal dung beetle revealed larger subunits of the LUC in the nocturnal species (Immonen et al., 2017). The nocturnal dung beetle species relies more heavily on polarized light (el Jundi et al., 2015), which could explain the need for more neurons that process this information and relay it to the LX. This in turn likely leads to a size-increase of the AOTU subunit that contains these cells. Following this line of argument, we predict that the nodular unit contains more neurons in the migratory Bogong moth, which implies that this area processes information crucially needed by this species. This is supported by the finding that in Monarch butterflies at least parts of this region contain neurons relaying visual compass information to the LX-bulbs (Heinze and Reppert, 2011; Heinze et al., 2013). In contrast, comparison of the same regions between migratory and non-migratory Monarch butterflies showed no differences in the small AOTU subunits, while revealing a decrease in the AOTU upper unit and a significant increase in the volume of the PB in experienced migrants (Heinze et al., 2013). This means that, overall, no differences were consistent across species that can be correlated with migratory behavior. Whether the smaller size of the gall in migratory Bogong moths in comparison to the Turnip moth indicates that the output pathway mediated via this region is less important for migratory behavior in general, cannot be solved at this point, as this region was not analyzed separately in the Monarch butterfly (Heinze et al., 2013).

### Potential Effects Due to Plasticity

One potentially confounding factor of the presented analysis is the different origin of used individuals: Bogong moths were wild-caught after their spring migration, Turnip moths were laboratory-raised, while Monarch butterflies had been freshly eclosed individuals (Heinze et al., 2013). Monarch butterflies were therefore age-matched, while individuals of both moth species were of unknown age, with potentially widely variable origin populations in case of the Bogong moth (Warrant et al., 2016). The consistent age of all Monarch butterfly individuals likely explains the considerably lower variability in the data for this species compared to the moths. Accordingly, the highest variability was found in the Bogong moth data, consistent with the least controlled sampling. The question therefore arises whether the species can be directly compared without taking into account age and experience effects of each population.

Age and experience indeed significantly influence volumes of brain regions in lepidopteran insects, including in the Monarch butterfly (Heinze et al., 2013). The most profound effects were found in *Heliconius* butterflies and were associated with the mushroom bodies. These structures showed remarkably large volume increases in wild-caught, experienced butterflies compared to young individuals or old individuals raised in captivity (Montgomery et al., 2016). Comparing old and young Monarch butterflies, a similar increase in overall neuropil volume was reported for the compass neuropils (Heinze et al., 2013), while grade shifts associated with extensive migratory experience were only found for the PB (larger) and the AOTU upper unit (smaller). These small changes in neuropil volume are the only experience-dependent changes reported for components of the “compass neuropils” to date. If similar effects exist in the examined moth species, they are expected to specifically have affected the Bogong moth brains (experienced individuals), likely amplifying any innate differences. As no large differences were revealed between the two moth species, effects due to migratory experience have most likely not been obscured by the differences in sampling. In fact, the only differences found between migratory Bogong moths and non-migratory Turnip moths were opposite to what would have been expected from comparing migratory and non-migratory Monarch butterflies (smaller PB and larger AOTU in migratory Bogong moths). Whether the sampling of the Bogong moth during their aestivating state, i.e., a state of dormancy, could explain these opposite than expected effects remains an open question. However, as the aestivation state is embedded between two migratory episodes during a Bogong moth’s life, any volume change associated with migration would have to be reversible between spring migrants, aestivating state and fall migrants, a degree of plasticity that has to date not been observed in insects, but which nevertheless provides an interesting subject for future studies.

### What Does It Take to Migrate?

Migrating over thousands of kilometers over unfamiliar terrain, equipped with a brain the size of a grain of rice seems a daunting endeavor. The question arises how the brain, in particular that of an insect, has to adapt in order to allow these journeys to be successful. In other words, what are the fundamental properties of a brain needed to guide migration? And why is there no strong evidence reflecting that behavior at the level of neuropil structure?

The challenges are both sensory and motor in nature. The brain has to ensure that the available information is used to extract a compass bearing as reliably as needed, while the motor control circuits have to ensure that the correct compass heading is faithfully maintained. If either one of those circuits, or the feedback between them, fails, the animal will not reach its goal and perish. In this respect, mistakes are much more devastating during migration than they are during opportunistic foraging. Given this strong selective pressure, migratory species can be expected to have invested in neural circuitry that allows their brains to perform course control more reliably than species that can get by with making more mistakes. However, other than being optimized for reliability, the basic circuits likely do not need to be fundamentally altered, as all animals need to compare their desired heading with their current heading and compensate any mismatch with steering movements. The only difference in migrants is that the desired heading is constant over time and does not change with each new situation.

How are the control circuits made sufficiently reliable to avoid failure over thousands of kilometers of travel? On the sensory side, migrants should rely on multiple sources of compass information that are integrated into a coherent estimate of current heading within a global reference frame. For different visual compass cues this integration has been shown physiologically in locusts (Kinoshita et al., 2007; Pfeiffer and Homberg, 2007; el Jundi et al., 2014) and Monarch butterflies (Heinze and Reppert, 2011). Beyond integrating several sources of information, it is also important how this information is processed. While this clearly asks for more physiological experiments, anatomical data also can provide first hints. Reliability can be achieved by more neurons carrying compass signals to the central steering control, in order to allow averaging and eliminating noise, or by more reliable synapses in the compass pathway. This would be in line with our finding that the small subunits of the AOTU, which provide links between the optic lobe and the CX, are larger in the Bogong moth, potentially housing more neurons or larger synapses.

Once a multisensory reference frame is established, we can expect further differences between migratory and non-migratory species. Even though a reference frame is useful for, and potentially required by, all species, migrants should have a reference frame that is either fixed, or regularly recalibrated. The systematic E-vector representation in the locust PB, found over many individuals, suggests a fixed reference frame for that species (Heinze and Homberg, 2007), whereas the changing offset between anatomy and physiology of the landmark-based head direction signal in *Drosophila* (Seelig and Jayaraman, 2015) indicates a flexible reference frame.

On the motor output side, similar arguments to those we made for the sensory inputs also apply and these narrow down the candidate regions housing migration-specific adaptations to the two output areas of the CX: the LAL and the gall (Heinze and Homberg, 2008; Heinze et al., 2013; Wolff et al., 2015). Whereas CX-activity directly modulates turning reflexes in walking cockroaches (Martin et al., 2015), nothing is known about the anatomical substrate of the neurons involved or whether similar direct coupling happens during flight behavior. It therefore remains to be shown which output pathway contains migration relevant adaptations. The smaller gall-volume we found in the Bogong moth might suggest that the gall-output pathway is less relevant for migration than for opportunistic foraging.

Finally, how is the intended heading fixed neurally? Unfortunately, we currently don’t know how any intended heading is encoded in an insect brain. Yet it is difficult to imagine that a fixed migratory bearing requires more neural capacity than a flexible one. Thus, these differences are unlikely to be found at the level of neuropils, but rather at the level of circuit connectivity.

### The Standard Atlases as Basis for Comparative Circuit Analysis

As the neural underpinnings of migration are likely found at the level of neurons and neural circuits, we have generated the average atlases of the two moth species not only for direct shape and volume comparisons, but to provide a tool to perform efficient circuit analysis. By registration of individually dye injected neurons from intracellular recordings into a common reference frame, these atlases will allow us to map, collect and directly compare neurons between the two species. Differences in the sizes of arborization fields, fiber paths, degree of neural overlap, and likely pathways of information flow can be examined in detail using this tool, similar to what has already been achieved for the desert locust (el Jundi et al., 2009a) and the Monarch butterfly (Heinze et al., 2013).

## Conclusion

In summary, our anatomical comparison of the regions likely to be involved in guiding compass navigation in migratory Bogong moths has revealed no major qualitative differences between migratory species and non-migratory species. This strongly suggests that the adaptations necessary to ensure successful migratory behavior are manifested at the level of neural circuits and will only be accessible via detailed physiological investigations. The current study aids this endeavor in two ways. First, the identified differences in neuropil volume in the nodular unit of the AOTU, the PB and the gall region of the LX, indicate promising target areas for electrophysiology both on the input and on the output side of the likely control circuits. Second, our new average-shape standard atlas provides an anatomical reference frame in which to embed all functional data obtained from the brain of the remarkable Bogong moth.

## Ethics Statement

All animal procedures were in compliance with the guidelines of the European Union (Directive 86/609/EEc). Approval of the study by an ethics committee was dispensable, because all experiments were on insects.

## Author Contributions

LV and SH designed the study, performed histological experiments, wrote the manuscript and designed figures. LV carried out 3D-reconstructions and data analysis. AKA carried out electrophysiology, histology, reconstruction and registration of single neurons. KP and BT wrote standardization routines and performed brain atlas generation. EW and KG established Bogong moth field sites, captured moths and co-designed the study. All authors critically read the article and contributed to its final version.

## Conflict of Interest Statement

The authors declare that the research was conducted in the absence of any commercial or financial relationships that could be construed as a potential conflict of interest.
