# Supplementary material for: Comparison of Navigation-Related Brain Regions in Migratory versus Non-Migratory Noctuid Moths
Source: Front Behav Neurosci. 2017 Sep 4;11:158. doi: 10.3389/fnbeh.2017.00158 (PMC5591330; doi:10.3389/fnbeh.2017.00158)
Supplement: Supplementary file 1 [file Data_Sheet_1.pdf]

Supplementary Material

Comparison of navigation-related brain regions in migratory versus non-migratory noctuid moths

Liv de Vries[1], Keram Pfeiffer[2], Björn Trebels[2], Andrea Adden[1], Ken Green[3], Eric Warrant[1], *Stanley Heinze[1]

*correspondence: Stanley.heinze@biol.lu.se

Supplementary Figures and Tables

1.1 Supplementary Figure

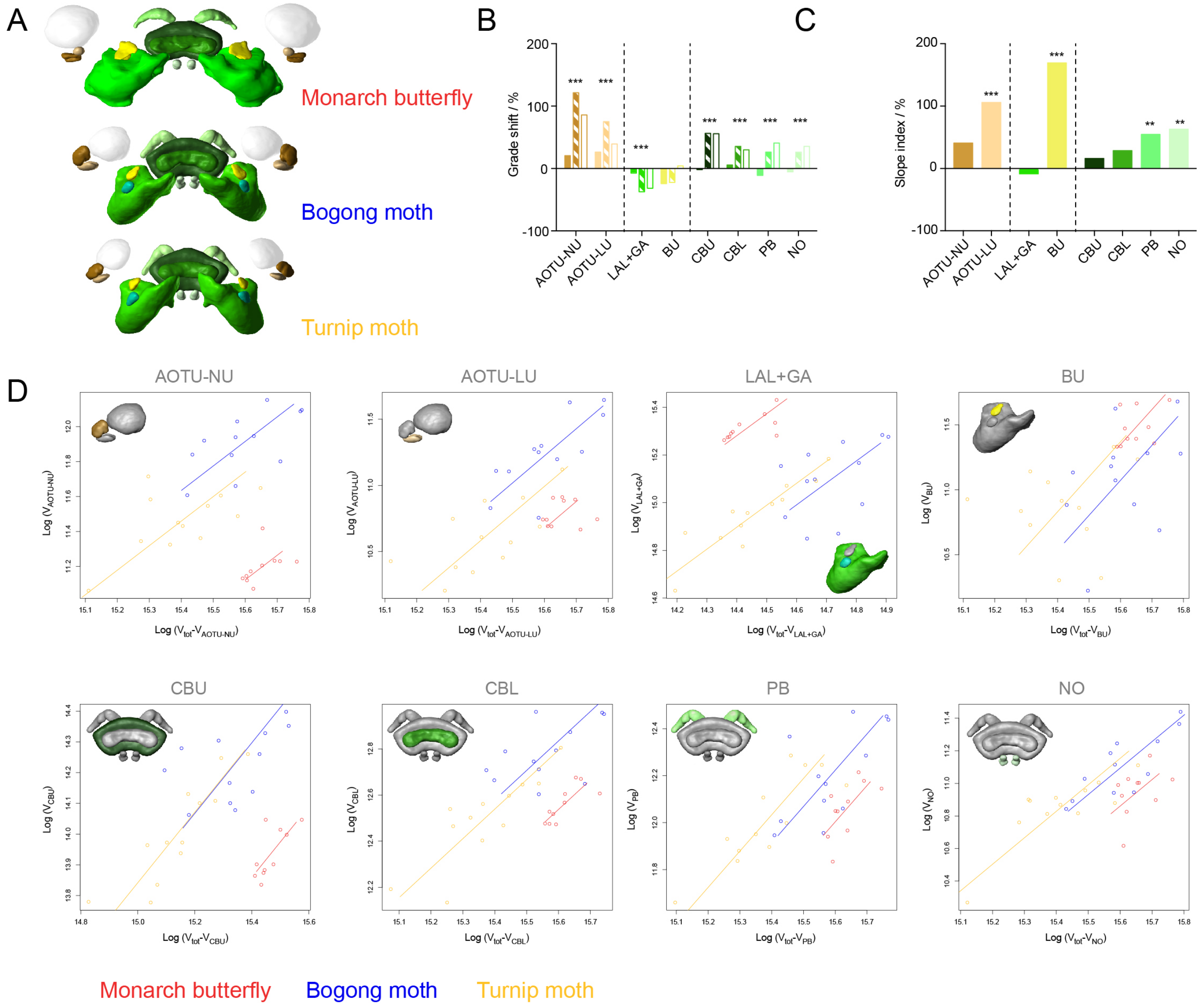

Figure S1: Results from standardized major axis regression analysis comparing the Bogong moth, the Turnip moth and the Monarch butterfly compass neuropils with upper unit of the anterior optic tubercle (AOTU-UU) *excluded* from analysis. **A,** Surface rendering of neuropils in each species**. B,** Analysis of grade shift indices (GSI) of all neuropils. *Solid bars*: Bogong moth – Turnip moth comparison (values > 0: Bogong moth bigger; values < 0: Bogong moth smaller); *hatched bars*: Bogong moth – Monarch butterfly comparison (values > 0: Bogong moth bigger; values < 0: Bogong moth smaller); *open bars*: Turnip moth – Monarch butterfly comparison (values > 0: Turnip moth bigger; values < 0: Turnip moth smaller). Asterisks indicate significance level of GSI. **C,** Analysis of slope index for all neuropils. Values larger than zero indicate steeper than isometric scaling. Asterisks illustrate significance level of deviations from isometric scaling. **D,** Regression analysis data; red, Monarch butterfly; blue, Bogong moth; yellow, Turnip moth. For each neuropil individual data-points are shown together with the best-fit common slope regression line. The inset depicts the respective neuropil for each graph highlighted in color. *Abbreviations*: AOTU, anterior optic tubercle; LU, lower unit; NU, nodular unit; CBL, lower division of the central body; CBU, upper division of the central body; BU, bulb; LAL, lateral accessory lobe; GA, gall; PB protocerebral bridge; NO, noduli.

1.2 Supplementary Tables

Table S1: Bogong moth (*A. infusa*) raw volume data in µm^3.

#	CBU	CBL	PB	NO	LAL	BU	GA	AOTU-UU	AOTU-LU	AOTU-NU
1	1 632 080	425 485	191 824	76 530	3 802 817	111 583	41 052	1 073 239	80 724	167 690
2	1 280 995	316 181	163 744	53 713	3 075 440	68 386	24 137	727 163	66 817	138 703
3	1 706 418	423 901	255 948	86 214	4 342 209	118 008	45 360	1 184 634	1 02 019	177 711
4	1 586 525	358 383	234 867	61 581	2 870 913	27 569	37 638	867 491	66 566	150 234
5	1 328 715	343 159	198 398	72 140	3 577 777	71 698	42 837	776 242	78 726	138 261
6	1 557 548	389 675	260 980	63 418	4 080 787	79 546	50 130	1 587 353	1 12 159	189 648
7	1 298 799	297 618	155 746	56 498	3 812 154	76 712	34 746	740 335	46 900	115 877
8	1 482 833	330 339	154 235	51 170	2 811 179	53 272	33 231	741 095	50 456	109 933
9	1 419 746	331 542	178 721	67 203	3 603 577	64 365	33 310	790 002	76 968	153 204
10	1 668 929	311 333	216 586	77 524	4 218 408	43 857	33 329	709 084	77 209	133 384
11	1 380 601	359 919	172 828	56 680	3 998 486	53 545	24 423	772 551	72 989	154 195
12	1 792 119	421 362	252 311	92 913	4 309 239	79 052	52 948	919 969	1 14 231	179 061

Table S2: Turnip moth (*A. segetum*) raw volume data in µm^3.

#	CBU	CBL	PB	NO	LAL	BU	GA	AOTU-UU	AOTU-LU	AOTU-NU
1	1 159 419	259 061	144 362	53 836	2 680 562	68 954	36 039	679 106	32 272	107 329
2	962 709	186 086	151 820	47 119	2 846 937	50 843	30 115	583 174	27 033	84 515
3	1 334 310	295 643	216 643	57 329	3 278 693	44 308	29 977	693 293	38 927	103 423
4	1 171 648	285 335	146 700	52 496	3 096 240	54 993	37 060	663 814	53 342	92 181
5	1 342 476	310 379	208 042	60 187	3 478 323	30 378	33 587	652 908	54 577	109 721
6	1 018 280	279 109	138 220	54 212	2 783 801	45 675	31 607	711 022	46 526	122 389
7	1 555 587	364 108	190 863	66 910	3 879 683	75 638	48 773	757 369	67 564	114 541
8	1 371 760	259 989	162 478	49 829	3 217 156	52 104	34 871	585 274	34 697	85 955
9	1 537 539	311 745	208 125	53 073	3 539 631	83 269	40 433	546 148	43 851	97 514
10	1 130 716	243 523	181 157	54 796	3 119 676	29 856	34 547	616 670	40 503	93 993
11	1 172 836	268 860	154 948	49 638	2 939 766	63 417	26 592	510 194	31 066	82 843
12	964 878	197 236	115 799	28 813	2 220 596	55 660	37 259	487 192	33 780	63 650

Table S3: Monarch butterfly (*Danaus plexippus*) raw volume data in µm^3 (from Heinze et al. 2013).

#	CBU	CBL	PB	NO	LAL+GA	BU	AOTU-UU	AOTU-LU	AOTU-NU	AOTU-SP
1	1 059 305	273 146	171 039	50 301	4 369 822	95 922	2 510 081	32 756	71 100	11 180
2	1 088 330	287 025	157 402	60 035	4 542 636	88 645	2 851 821	41 140	73 507	13 674
3	1 022 236	275 921	182 305	40 807	4 312 863	115 267	2 421 278	32 683	67 503	11 350
4	1 220 291	319 984	201 720	71 002	4 569 813	96 935	2 692 202	44 758	75 522	9 059
5	1 263 928	298 598	188 175	61 318	5 025 502	119 450	2 659 709	35 587	75 152	10 782
6	1 257 741	297 882	178 063	60 069	4 327 677	116 285	2 656 816	39 128	90 923	14 197
7	1 072 378	261 147	170 798	61 493	4 401 317	88 627	2 326 968	37 140	64 336	17 390
8	1 200 738	318 079	194 467	54 058	4 740 902	85 518	2 613 147	34 040	75 366	8 866
9	1 090 315	261 728	137 841	55 498	4 302 766	83 609	2 302 842	35 131	69 215	11 084
10	1 050 844	262 095	153 306	59 286	4 253 526	83 361	2 370 985	29 602	68 306	16 667
11	1 059 305	273 146	171 039	50 301	4 369 822	95 922	2 510 081	32 756	71 100	11 180
12	1 088 330	287 025	157 402	60 035	4 542 636	88 645	2 851 821	41 140	73 507	13 674
